# Prevalence of Low Back Pain and Associated Risk Factors among Saudi Arabian Adolescents: A Cross-Sectional Study

**DOI:** 10.3390/ijerph191811217

**Published:** 2022-09-07

**Authors:** Ahmed S. Alhowimel, Reem M. Alfaifi, Abdulkarim A. Alluhaybi, Majed A. Alanazi, Khalid M. Alanazi, Nouf S. Almathami, Sarah H. Almedhwah, Abdullah A. Almuayli, Aqeel M. Alenazi, Mohammed M. Alshehri, Bader A. Alqahtani, Faris Alodaibi

**Affiliations:** 1 Department of Health and Rehabilitation Sciences, Prince Sattam Bin Abdulaziz University, Al-Kharj 16278, Saudi Arabia; 2 Al-Rass General Hospital, Al-Rass 58883, Saudi Arabia; 3 Al-Hayat National Hospital, Madinah 42316, Saudi Arabia; 4 Comprehensive Rehabilitation Center for People with Disabilities, Arar 73552, Saudi Arabia; 5 National Guard Health Affairs, Western Section, Riyadh 11426, Saudi Arabia; 6 Saudi German Hospital, Riyadh 62451, Saudi Arabia; 7 King Khalid Hospital, Al-Kharj 16271, Saudi Arabia; 8 Physical Therapy Department, Jazan University, Jazan 82775, Saudi Arabia; 9 Department of Rehabilitation Science, King Saud University, Riyadh 11451, Saudi Arabia

**Keywords:** low back pain, prevalence, adolescent

## Abstract

Low back pain is the most prevalent musculoskeletal condition. Studies on adolescent low back pain are scarce, with no research to determine its prevalence in Saudi Arabia. This study aimed to assess the prevalence and associated risk factors of low back pain in Saudi Arabian adolescents. This cross-sectional study was conducted among Saudi Arabian high school students, which included demographic data, medical and low back pain history. The completed survey by 2000 participants showcased 57.9% of students experiencing low back pain in the last 12 months. This included 31.2% men and 26.7% women. This study found a link between low back pain and age, clinical symptoms of diabetes, heart disease, lung disease, thyroid disease, arthritis, and back surgery, a family history of low back pain, as well as smoking. There is substantial prevalence of low back pain in Saudi Arabian adolescents. This study identified several modifiable and non-modifiable risk factors stemming from adolescent low back pain.

## 1. Introduction

Lower back pain (LBP) is the most prevalent condition globally [1]. A study conducted by Vos et al. [2] reported that LBP prevalence was higher than that of HIV, road traffic accidents, and lung cancer. In addition, Wu et al. [3] reported the worldwide point prevalence rate of LBP to be 7.5%.

Three sociodemographic factors were associated with the prevalence of LBP: occupational nature, age, and gender. Adults are far more prone to LBP than children, with peak incidence occurring at 35–55 years old. According to Wu et al. [3], females have a higher LBP prevalence than males. However, other studies found a similar prevalence of LBP among men and women [4,5]. In addition, there are different prevalence rates of LBP in different countries [3]. The differences are associated with sociodemographic characteristics and geographical location. Additionally, Hartvigsen et al. [6] stated that high-income countries have a higher risk of LBP, with the prevalence rates of LBP in rich and poor countries at 30% and 18%, respectively.

The Kingdom of Saudi Arabia has become one of the countries with a high prevalence of LBP, due to rapidly changing economic and social aspects that created increasing public health challenges [7]. Although there is a lack of consensus and the numbers vary, some studies have indicated the Saudi Arabian prevalence rate of LBP to be between 63.8% and 89% among different professional workers [8]. Conversely, a recent community-dwelling study suggested that the prevalence rate of LBP in Saudi Arabia is 27.9% [4].

Several studies have been conducted to determine the prevalence of and risk factors for LBP among students. For example, a recent study in Malaysia showed that the incidence of musculoskeletal disorders among Malaysian medical students was relatively high (46%) [9]. A similar study conducted on medical students at Saudi Arabia’s Jazan University reported a high prevalence of musculoskeletal disorders, with risks increasing due to a history of trauma, depression, and psychosomatic symptoms [10]. However, no studies have been conducted to determine the prevalence and associated risk factors of LBP in high school students. Early screening and detection of LBP may lead to better outcomes and improvements in younger age. Therefore, this study aimed to explore the prevalence and associated risk factors of LBP in Saudi Arabian adolescents.

## 2. Methods

This is a cross-sectional study on high school students from five different Saudi Arabian regions, which account for nearly 70% of the country’s total population (Riyadh, Jeddah, Medina, Asir, and Arar). The study investigated the prevalence of LBP in male and female high school students using simple random sampling technique. Other objectives included exploring any associated risk factors for LBP among participants and the impact on their lifestyle. Data were collected electronically using a detailed sectional questionnaire from November 2021 to April 2022.

The sample size was calculated using a statistical formula for a cross-sectional study [11]. We assumed the prevalence rate of LBP to be approximately 50% as the safest choice with a 95% confidence interval for single population proportion and 5% precision. The minimum sample size was 768, multiplied by the number of domains, which was two based on the two sex groups (male and female). This yielded a sample size of 1536.

Inclusion criteria:

Participants were included if they were,

- Saudi Arabian
- Between 14 and 18 years old.

Exclusion criteria:

Participants were included if they have,

- Congenital musculoskeletal or neurological disorders.
- Recent injuries.

This study was approved by the Ethical Committee of Applied Medical Sciences, Prince Sattam Bin Abdulaziz University, Al Kharj, Kingdom of Saudi Arabia (No.: RHPT/021/014). The researchers sought consent from the students before they responded to the questionnaire.

A closed-ended, self-reported Arabic questionnaire was used to collect data (Supplementary Materials). The student must respond to all questions. In order to improve the clarity and validity of the questionnaire items, 10 volunteers of similar age were asked to evaluate their clarity, relevance, and comprehension. The final version was modified to incorporate an explanation of medical words such as asthma and COPD. The questionnaire was divided into two sections:

- The first section included socio-demographic characteristics (age in years, sex as males or females, body mass index (BMI) calculated by dividing weight in kg on squared height in centimeter, and smoking status as yes or no) and the presence of any comorbidity as yes or no (diabetes mellitus, cardiovascular disease, respiratory diseases (asthma, COPD, apnea), thyroid diseases, arthritis, scoliosis, and back surgery). Family history of back pain was obtained from participants. Physical activity was reported for all participants as follows: less than an hour/week, 2–3 h/week, 3–4 h/week, more than 4 h/week or never.
- The second section assessed pain in the last 12 months and the last seven days. In this section, participants were enquired about the presence of pain, the impact of pain on leisure and physical activity, their frequency of hospital visits, as well as the frequency of missing school. These questions were answered with YES/NO as follows “Have you ever had lower back problems (pain or uncomfortable feeling)?”, “Have you ever visited the hospital because of lower back problems?”, and “Did back problems cause you to change your lifestyles or duties?” Another question was related to the length of time of back pain as follows “What is the total length for which you have suffered from back problems in the last 12 months?” The answered included 0 days, 1–7 days, 8–30 days, more than 30 days, but not daily or daily. If the answer was not 0, the participants were prompted to other questions related to activities in the past 12 months as follows: “Has your daily activity decreased during the past 12 months due to back problems?”, “Work-related activities, whether at home or outside”, and “leisure activities”. The answers were recorded as YES/NO. Further questions were asked as follows: “What is the total length of time you lost your work or recreational activities due to back problems?” The answers were recorded as 0 days, 1–7 days, 8–30 days or more than 30 days. A YES/NO question related to seeking health care due to back pain was asked as follows: “Have you ever consulted a doctor, physiotherapist or chiropractor because of back problems?” Back pain over 7 days was recorded as follows using a YES/NO question as follows “Did you suffer from back problems during the past week (7 days)?” Finally, back pain severity was examined using numeric rating scale from 0 “no pain” to 10 “severe pain”.

The participants in this study included high school students from various regions throughout Saudi Arabia. The researchers (i.e., with physiotherapy degrees) visited the schools and distributed the questionnaire among students who were willing to participate in the study. In other areas, students were invited to participate in an electronic survey, distributed via the social media networks of Twitter, Telegram, and WhatsApp.

The data were analyzed using the Statistical Package for Social Sciences (SPSS version 26, IBM, Armonk, NY, USA). The primary outcomes were the presence of LBP in the last 12 months, and the last seven days, shown as means with standard deviations. The chi-square test was used to assess the association between categorical variables, and the Mann–Whitney test was used for continuous variables. Multivariable binary logistic regression was used to assess the presence of LBP against associated risk factors. Independent variables (risk factors) included age, sex, BMI, smoking status, and comorbidities (i.e., diabetes mellitus, cardiovascular disease, respiratory diseases (asthma, COPD, apnea), thyroid diseases, arthritis, scoliosis, and back surgery) as well as family history of back pain. Dependent variable was the presence of back pain. Statistical significance was set at *p* ≤ 0.05.

## 3. Results

In total, 2000 participants completed the survey. The prevalence rate of LBP in the last 12 months was 57.9% (n = 1158). Hospital visits for LBP were reported by 11.3% (n = 225) of the participants and 10% (n = 199) of them visited doctors, physiotherapists, or chiropractors for LBP. Additionally, 19.1% (n = 382) of the participants had reduced their overall activity and 18.9% (n = 377) of them reduced only their leisure activities due to LBP.

The prevalence rate of LBP in the last seven days was 28.5% (n = 570), with 12.85% males (n = 257) and 15.6% females (n = 313); the average pain severity for all the participants was reported to be 3.22 ± 2.93.

Table 1 shows the total duration for which they experienced LBP during the last 12 months and the total duration that LBP had prevented them from going to school and performing general activities during the last 12 months.

Table 2 shows the demographic and clinical characteristics of the participants. Among these risks, age, gender, and clinical manifestations of diabetes mellitus, cardiovascular disease, respiratory diseases (such as asthma, asthma, COPD, apnea), thyroid diseases, arthritis, and back surgery, LBP family history, and lifestyle habits of smoking, physical activity, electrical device usage, and coffee consumption, were significantly different (*p* < 0.05) among participants with LBP compared to those without. In contrast, BMI showed no significant difference (*p* = 0.062) between participants with and without LBP.

Table 3 shows the association between LBP and clinical variables, with odds ratio and 95% CI. Participants with respiratory diseases were twice as likely to have LBP, than those without. Participants with thyroid diseases were eight times more likely to have LBP than those without the diseases. Similarly, participants with arthritis were seven times more likely to have LBP than those without. Although the majority of the participants were male, the prevalence rate of LBP in females was higher at 74% compared to males at 48%. Additionally, older participants were more likely to have LBP than younger participants, and smokers were twice as likely to have LBP than non-smokers.

## 4. Discussion

This study aimed to determine the prevalence of LBP among Saudi Arabian adolescents and identify its associated risk factors. The participant LBP prevalence rate for the last 12 months was 57.9%, with a higher rate for females than for males. The prevalence rate in the last seven days was 28.5%.

There were significant differences among all participants experiencing LBP as well as those who did not based on age, clinical manifestation of diabetes mellitus, cardiovascular disease, respiratory diseases, thyroid diseases, arthritis, back surgery, LBP family history, and smoking. In contrast, there was no significant difference in BMI between the participants with and without LBP.

A previous study conducted by Brazil’s Silva et al. [12] reported a high prevalence rate of LBP among students aged 12–15 years, at 57% (n = 195); 60% for females (n = 125) and 53% for males (n = 70). Another study of 1608 adolescents (825 females and 783 males) by Western Australia’s O’Sullivan et al. [13] showed the prevalence rate of LBP at 46.5%. In this study, gender primarily affected back pain, where females were more likely to have LBP than males. Most previous studies reported an equal LBP incidence rate for male and female, with a few studies reporting a higher incidence of LBP in females. In a study of 600 adolescents conducted in Bauru, Sao Paolo, Brazil, Bento et al. [14] found that 60% of female adolescents experienced LBP compared to 39.1% of males. In addition, another cohort study from Japan (n = 31,429) revealed that adolescent female students were more likely to suffer from LBP than the males [15].

This study’s LBP prevalence rate was 2.7% (n = 53) at 14 years old, increasing to 21% (n = 420) at 18 years old. Coenen et al. [16] found that individuals’ LBP prevalence rate from 17 to 22 years of age increased from 32% to 45%.

This study identified a relationship between LBP and a family history of LBP. A cross-sectional study conducted in Hong Kong found that a family history of back pain posed significant risks of spinal degeneration, as assessed by MRI [17].

In previous studies, LBP in adult patients was related to whether the patients had a normal or high BMI [18,19]. This result was similar to that of a cohort study that assessed the association between overweight adolescents and LBP [20]. In contrast, the current study failed to find an association between BMI and LBP.

In the present study, there was a significant relationship between LBP and diabetes mellitus, cardiovascular disease, respiratory diseases (asthma, COPD, apnea), thyroid diseases, arthritis, and back surgery. The exact pathophysiological causes of most musculoskeletal disorders remain unclear. Patients with diabetes mellitus suffer from connective tissue disorders, owing to collagen deposition, which alters their structural matrix and mechanical properties. Here, diabetes posed as a risk for LBP. Several other studies have found a link between hyperglycemia and biochemical events, that may underlie intervertebral disc degeneration. Previous studies have suggested that LBP is more prevalent in patients with diabetes mellitus [13,21].

Similarly, cardiovascular disease proved risky for LBP according to our study. Many previous studies have assumed that patients with musculoskeletal pain and cardiovascular diseases suffer from higher pain and disability than patients with only chronic LBP [22,23]. Patients with cardiovascular diseases are difficult to treat due to the consequences of these diseases, including limited exercise tolerance and dyspnea. This result was in line with that of a systematic review by Oliveira et al., in 2019, which also found a relationship between LBP and cardiovascular diseases [21].

In the present study, there was a significant relationship between respiratory disease and LBP. Patients with respiratory diseases were twice as likely to develop LBP and experience more severe pain. Previous studies have suggested that the muscle response of breathing is attached to the lumbar vertebra, and several studies have found that respiratory diseases are associated with LBP [24,25]. A systemic review by Beeckmans et al., (2016) found a significant correlation between dyspnea, asthma, different forms of allergies, and respiratory infections with LBP [26].

This study also found a significant relationship between thyroid diseases and LBP. They increase the likelihood of developing LBP by eight times. A case report by Sowmini et al. (2013) found a relationship between hypothyroidism and LBP. Another study by Elenius et al. (2021) suggested a relationship between hyperthyroidism and LBP [27,28].

In clinical settings, arthritis is one of the most prevalent conditions besides chronic LBP [29]. Osteoarthritis is the most common arthropathy, which is a degenerative joint disease that involves joint cartilage and remodeling. This study suggested a relationship between LBP and arthritis, and its effect on the severity of pain; patients with arthritis were seven times more likely to have LBP. Several studies have found a correlation between LBP and arthritis [30,31].

The results of this study suggest that smoking has a significant effect on LBP; participants who smoked were twice as likely to experience LBP. Frymoyer et al., were the first to postulate that people with back pain had a higher prevalence of coughing and that mechanical stress caused by coughing might be related to LBP [32]. Several studies have investigated the association between LBP and smoking status. Shaw et al. only studied male participants with acute LBP, and discovered the increasing likelihood of chronic LBP due to cigarette smoking, along with psychological factors [33]. Furthermore, after controlling for numerous sociodemographic characteristics, Zvolensky et al. discovered that people addicted to nicotine were almost twice as likely to report chronic neck or back pain [34]. Schembri et al. revealed that both smoking status and nicotine dependency were independently associated with an elevated incidence of chronic LBP [35].

In this present study, there was a relationship between the level of activity and LBP. Several studies highlighted that physical activity in childhood had an impact on back pain reporting in early adolescence. Wedderkopp et al. suggested that high physical activity levels may reduce the odds of future back pain [36]. Rahman et al. also found that leisure-time physical activity may reduce the risk of chronic LBP by 11% to 16% [37]. Cormac et al. found that individuals with chronic LBP had a lower level of physical activity [38].

In the present study, body mass index (BMI) was not associated with LBP. However, multiple studies on adolescent populations have reported a significant association between BMI and the presence of LBP [39,40]. It should be noted that we used a self-reported measure of BMI, which lacks accuracy and might be misleading in classifying obesity in adolescents according to Karchynskaya et al. (2020) [41].

The study’s generalizability may have been enhanced by the fact that samples were recruited from diverse locations of Saudi Arabia, which account for more than 70% of the population. LBP in adolescents need the attention of all stakeholders, including educators, legislators, healthcare providers, and parents. Further research is necessary to understand which lifestyle behaviors are associated to LBP and whether modifying lifestyle behaviors have an influence on LBP in adolescents.

This study had several limitations. First, an unequal number of male and female participants engaged in this study. However, this might be reflecting the higher population of men than women Second, this research was not observational, and did not conduct a separate variable examination to determine their longitudinal pathophysiology and relationship with LBP. Due to the nature of exploratory studies, we were not able to use extensive methodologies to assure the accurate relationship between LBP and other risk factors. Future studies are needed to include back pain specialists in the interview and use causality design to overcome limitations of this study. Despite these limitations, the study demonstrated a substantial prevalence of LBP among Saudi Arabian adolescents, which was not previously studied. Hence, this necessitates further investigation to comprehend its prevalence and complex relationships among adolescents.

## 5. Conclusions

LBP was highly prevalent among Saudi Arabian adolescents with more occurrence among females and older adolescents. Despite that most LBP cases were short with minimal impact, a small percentage of LBP cases have stronger impact on the adolescents’ activity and could lead to disability. Number of modifiable and nonmodifiable risk factors have been identified including smoking, physical activity, diabetes mellitus, cardiovascular disease, respiratory diseases, thyroid diseases, arthritis, LBP family history, and back surgery.

## Figures and Tables

**Table 1:** Total duration of LBP and activity limitation during the last 12 months.

**Duration of LBP during the Last 12 Months**	
0 days (number, %)	192 (9.6%)
1–7 days	461 (23.1%)
8–30 days, but not every day	341 (17.1%)
Daily	164 (8.2%)
Total	1158 (57.9%)
**Total duration that LBP Prevented Activity during the Last 12 Months**	
0 days (number, %)	410 (20.5%)
1–7 days	390 (19.5%)
8–30 days, but not every day	101 (5.1%)
Daily	65 (3.3%)
Total	966 (48.3%)

**Table 2:** Demographics and clinical characteristics of participants.

Factors	Back Pain, n = 1158	No Back Pain, n = 842	* *p*-Value
** BMI (Mean ± SD)	26.08 ± 65.5	40.09 ± 464.2	0.062
**Age**			0.001
14 years (n = 104)	n = 53, (2.7%)	n = 51, 2.6%	
15 years (n = 207)	n = 103, (5.1%)	n = 104, 5.2%	
16 years (n = 524)	n = 263, (13.2%)	n = 261, 13.1%	
17 years (n = 562)	n = 319, (16.0%)	n = 243, 12.2%	
18 years (n = 603)	n = 420, (21.0%)	n = 183, 9.2%	
**Gender**			0.001
Male (n = 1278)	n = 624 (31.2%)	n = 654 (32.7%)	
Female (n = 722)	n = 534 (26.7%)	n = 188 (9.4%)	
**LBP Family history**			0.001
Yes (n = 376)	n = 295 (14.8%)	n = 81 (4.1%)	
No (n = 1624)	n = 863 (43.2%)	n = 761 (38.1%)	
**Chronic disease**			**0.001**
Diabetes mellitus (n = 31)	n = 14 (0.7%)	n = 17 (0.9%)	0.02
Cardiovascular disease (n = 10)	n = 4 (0. 2%)	n = 6 (0.3%)	0.03
Respiratory diseases (asthma, COPD, apnea) (n = 88)	n = 65 (3.3%)	n = 23 (1.2%)	0.02
Thyroid diseases (n = 12)	n = 11 (0.6%)	n = 1 (0.05%)	0.03
Arthritis (n = 30)	n = 27 (1.4%)	n = 3 (0.15%)	0.00
Scoliosis (n = 5)	n = 4 (0.2%)	n = 1 (0.05%)	0.01
Back surgery (n = 9)	n = 9 (0.5%)	n = 0	0.0
Nothing (n = 1815)	n = 993 (50.7%)	n = 822 (39.75%)	0.01
**Smoke**			0.012
Yes (n = 1158)	n = 87 (4.4%)	n = 1071 (53.6%)	
No (n = 842)	n = 40 (2.0%)	n = 802 (40.1%)	
Pain severity mean ± SD	3.22 ± 2.93		

* *p*-value was based on the chi-square test for categorical variables or the Mann–Whitney test for continuous variables. ** BMI: Body Mass Index.

**Table 3:** Multivariate logistic regression to assess LBP against clinical variables.

Variable	* OR (95%CI)	*p*-Value
Diabetes mellitus	0.64 (0.31, 1.3)	0.23
Cardiovascular disease	0.52 (0.14, 1.8)	0.32
Respiratory diseases (asthma, COPD, apnea)	2.2 (1.3, 3.6)	0.00
Thyroid diseases	8.66 (1.11, 67.2)	0.03
Arthritis	7.08 (2.14, 23.44)	0.00
Age		
14 years	0.42 (0.26, 0.67)	0.00
15 years	0.45 (0.31, 0.63)	0.00
16 years	0.56 (0.4, 0.7)	0.00
17 years	0.66(0.51, 0.8)	0.002
18 years	** 0	
LBP Family history		
Yes	2.76 (2.09, 3.6)	0.000
Smoke		
Yes	1.92 (1.2, 2.9)	0.02

* OR: Odds Ratio. ** Reference category.

## Data Availability

The data presented in this study are available on request from the corresponding author.

## Supplementary Materials

The following supporting information can be downloaded at: https://www.mdpi.com/article/10.3390/ijerph191811217/s1. Survey questionnaire.
