# Supplementary material for: Prevalence of Low Back Pain and Associated Risk Factors among Saudi Arabian Adolescents: A Cross-Sectional Study
Source: Int J Environ Res Public Health. 2022 Sep 7;19(18):11217. doi: 10.3390/ijerph191811217 (PMC9517652; doi:10.3390/ijerph191811217)

Demographics

Region:

- Riyadh
- Mecca
- Medina
- Tabuk
- Northern Border
- Asir
- Al Baha
- Najran
- Jizan
- Al-Jawf
- Hail
- Qassim
- Eastern

Gender:

- Male
- Female

Age:

- 14

- 15
- 16
- 17
- 18

Weight kg :………

Length cm :………

Do you suffer from the following diseases? please choose all options

- Chronic disease
- Diabetes mellitus
- Cardiovascular disease
- Respiratory diseases (asthma,COPD,apnea)
- Thyroid diseases
- Arthritis
- Scoliosis
- Back surgery
- Nothing

Is there a family history of low back pain?

- Yes
- No

Do you smoke?

- o Yes
- o No

How long have you been physically active?

- o Less than an hour/week
- o 2-3 hours/week
- o 3-4 hours/week
- o More than 4hours/week
- o Never

Low Back Pain Assessment

1) Have you ever had lower back problems (pain or uncomfortable feeling)?

- o Yes
- o No

2) Have you ever visited the hospital because of lower back problems?

- o Yes
- o No

3) Did back problems cause you to change your lifestyles or duties?

- Yes
- No

4) What is the total length for which you have suffered from back problems in the last 12 months?

- 0 days
- 1-7 days
- 8-30 days
- More than 30 days, but not daily
- Daily

If the answer to question 4 is zero days, do not answer questions 5 and 8

5) Has your daily activity decreased during the past 12 months due to back problems?

a) Work-related activities, whether at home or outside

- Yes
- No

b) leisurel activities

- Yes
- No

6) What is the total length of time you lost your work or recreational activities due to back problems?

- 0 days
- 1-7 days
- 8-30 days
- More than 30 days

7) Have you ever consulted a doctor, physiotherapist or chiropractor because of back problems?

- Yes
- No

8) Did you suffer from back problems during the past week (7 days)?

- Yes
- No

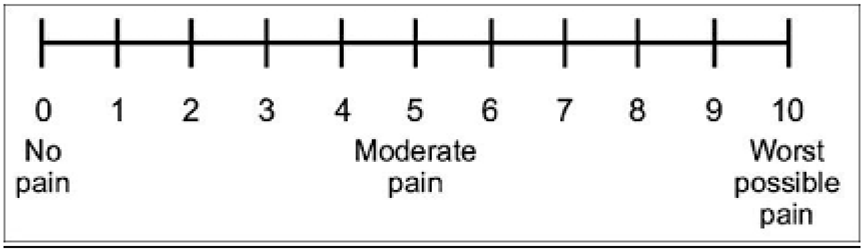

Supplement: Supplementary file 1 [file ijerph-19-11217-s001.zip › ijerph-1879567-supplementary.pdf]
